# Hemostatic Resuscitation in Trauma-Induced Coagulopathy: A Comprehensive Narrative Review

**DOI:** 10.3390/medicina62071263

**Published:** 2026-06-30

**Authors:** Matteo Matteucci, Bruno Cirillo, Francesco Brucchi, Fabio Suadoni, Antonio Pesce, Daniele Giuliani, Alessandro Spizzirri, Vincenzo Napolitano, Marta Micheli, Gianlorenzo Dionigi, Roberto Cirocchi

**Affiliations:** 1 Department of General Surgery, University of Milan, 20122 Milan, Italy; matteo.matteucci@unimi.it; 2 Department of Surgery, Sapienza University, 00161 Rome, Italy; bruno.cirillo@uniroma1.it; 3 Department of General Surgery, Istituto Auxologico Italiano, Istituto di Ricovero e Cura a Carattere Scientifico (IRCCS), 20145 Milan, Italy; francesco.brucchi@unimi.it (F.B.); g.dionigi@auxologico.it (G.D.); 4 Section of Legal Medicine, University of Perugia, 06123 Perugia, Italy; fabio.suadoni@unipg.it; 5 Department of Surgery, Azienda Unità Sanitaria Locale (AUSL) Ferrara, 44121 Ferrara, Italy; antonio.pesce@unife.it; 6 Department of Colorectal and General Surgery, Azienda Ospedaliera di Terni, 05100 Terni, Italy; d.giuliani@aospterni.it (D.G.); a.spizzirri@aospterni.it (A.S.); v.napolitano@aospterni.it (V.N.); 7 Department of Immunohaematology and Trasfusion, Azienda Ospedaliera di Terni, 05100 Terni, Italy; marta.micheli@aospterni.it; 8 Department of General Surgery, University of Perugia, 06123 Perugia, Italy

**Keywords:** trauma-induced coagulopathy, acute traumatic coagulopathy, TIC, hemostatic resuscitation, damage control resuscitation, viscoelastic testing, thromboelastometry, trauma guidelines for major bleeding

## Abstract

*Background and Objectives*: Traumatic hemorrhage remains the leading cause of preventable death following major injury, with most hemorrhage-related fatalities occurring within the first hours after trauma. During this early phase, trauma-induced coagulopathy (TIC) frequently develops as an independent pathophysiological response, affecting up to one-third of severely injured patients and being strongly associated with increased morbidity and mortality. Over the past two decades, TIC has been recognized as a complex endogenous process rather than a simple consequence of dilution, hypothermia, or acidosis, prompting a paradigm shift in early trauma resuscitation. *Materials and Methods*: This narrative review analyzes the current literature on the pathophysiology of TIC and the evolution of hemostatic resuscitation strategies. Key topics include the mechanisms underlying early coagulopathy, its clinical impact, and the evidence supporting contemporary therapeutic approaches. Published data on balanced transfusion strategies, whole blood transfusion, fibrinogen replacement, cryoprecipitate, prothrombin complex concentrates, tranexamic acid and viscoelastic-guided resuscitation were reviewed, along with relevant international guidelines. *Results*: Emerging evidence supports early, balanced, and targeted hemostatic resuscitation to mitigate the effects of TIC and improve outcomes in bleeding trauma patients. Balanced transfusion ratios, prompt correction of fibrinogen deficiency, early antifibrinolytic therapy and selective use of coagulation factor concentrates have been associated with reduced transfusion requirements and improved survival. Viscoelastic testing enables rapid, individualized assessment of coagulation abnormalities, although its availability and implementation remain inconsistent across trauma systems. *Conclusions*: Early recognition and aggressive, structured management of trauma-induced coagulopathy are essential to reduce preventable deaths from traumatic hemorrhage. While advances in hemostatic resuscitation have improved outcomes, significant challenges remain in standardizing treatment protocols and expanding access to viscoelastic diagnostics. Ongoing research and system-level optimization are needed to further refine and disseminate evidence-based strategies for the management of TIC.

## 1. Introduction

Traumatic hemorrhage remains a major global health burden and continues to account for a substantial proportion of early deaths following severe injury. The temporal distribution of trauma mortality is characterized by a marked concentration of deaths within the first minutes to hours after injury, with hemorrhage representing the leading cause of potentially preventable mortality [1]. Within this narrow therapeutic window, the prompt recognition and treatment of coagulopathy are critical, as delays in correcting hemostatic derangements are strongly associated with increased mortality. Historically, trauma-associated coagulopathy (TIC) was largely attributed to iatrogenic factors, including hemodilution, hypothermia, and acidosis occurring during resuscitation [2]. However, evidence accumulated over the past 15 years has clearly demonstrated that TIC is driven by a complex interplay of shock and hypoperfusion, endothelial dysfunction, activation of the protein C pathway, hyperfibrinolysis, platelet dysfunction and acute dysregulation of the coagulation cascade [3,4]. Because TIC is present on hospital admission in approximately one-quarter to one-third of severely injured patients, it has become a central focus in the evolution of modern trauma resuscitation strategies [5]. The emergence of hemostatic resuscitation represents a fundamental paradigm shift in trauma care. Rather than relying on large volumes of crystalloids and delayed blood product administration, contemporary management emphasizes early and balanced transfusion of plasma, platelets and red blood cells, along with rapid correction of fibrinogen deficiency and selective use of coagulation factor concentrates [6,7]. These strategies are integrated with adjunctive measures such as permissive hypotension and damage control surgery, which aim to limit ongoing bleeding and physiological exhaustion until definitive hemostasis can be achieved. In parallel, viscoelastic hemostatic assays, including rotational thromboelastometry (ROTEM) and thromboelastography (TEG), have assumed an increasingly prominent role in guiding resuscitation, as they provide real-time, functional assessment of clot formation, strength, and fibrinolysis [7]. This narrative review synthesizes current knowledge regarding the pathophysiological mechanisms of trauma-induced coagulopathy, available diagnostic modalities and the therapeutic components of hemostatic resuscitation. It also examines the strength of the evidence supporting these approaches, including findings from major randomized controlled trials, and reviews recommendations from contemporary international trauma guidelines [8,9]. By integrating mechanistic insights with clinical data, this review aims to provide a comprehensive overview of modern hemostatic resuscitation and its implications for improving survival in patients with severe traumatic injury.

## 2. Materials and Methods

This review was conducted as a narrative synthesis of the contemporary literature on trauma-induced coagulopathy and hemostatic resuscitation. A targeted literature search was performed using PubMed/MEDLINE and Scopus to identify relevant publications. The literature search was initiated in January 2025 and completed in April 2026. Only articles published in English were considered. The search strategy combined predefined keywords including: “trauma-induced coagulopathy”, “acute traumatic coagulopathy”, “TIC”, “traumatic hemorrhage”, “hemostatic resuscitation”, “damage control resuscitation”, “balanced transfusion”, “plasma-to-red blood cell ratio”, “whole blood transfusion”, “fibrinogen deficiency”, “fibrinogen concentrate”, “cryoprecipitate”, “tranexamic acid”, “viscoelastic testing”, “ROTEM”, “TEG”, and “trauma guidelines for major bleeding”. Boolean operators (AND/OR) were applied to refine the search strategy.

The review included randomized controlled trials, prospective and retrospective observational cohort studies, trauma registry analyses and international guideline documents considered relevant to contemporary trauma resuscitation practice. Particular attention was given to landmark trials evaluating transfusion strategies and antifibrinolytic therapy in trauma patients, including CRASH-2 [7], CRASH-3 [10], PROPPR [11], PAMPer [12], and COMBAT [13], as well as to the European Guideline on the Management of Major Bleeding and Coagulopathy Following Trauma [8]. Studies were selected based on their clinical relevance, methodological quality, and contribution to the understanding of trauma-induced coagulopathy, viscoelastic hemostatic testing, fibrinogen replacement strategies, balanced component therapy, whole blood transfusion, and coagulation factor concentrates. Case reports, conference abstracts without full-text availability, non-English publications, studies focused exclusively on non-traumatic coagulopathy, and articles judged to provide limited relevance to the topic were excluded. Additional articles were identified through manual screening of the reference lists of key publications.

Given the narrative nature of this review, no formal systematic review methodology, meta-analysis, or structured risk-of-bias assessment was performed.

## 3. Pathophysiology of Trauma-Induced Coagulopathy

**Figure 1:**
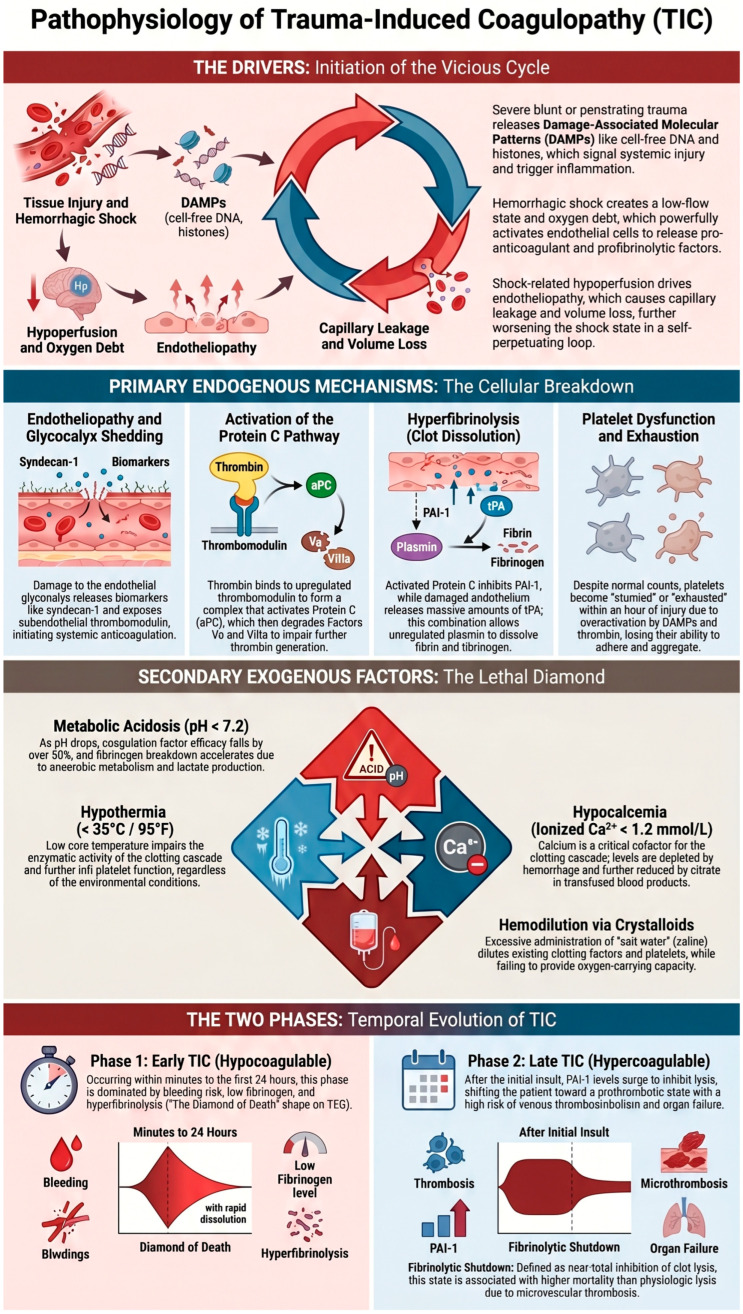
Pathophysiology of TIC. Severe trauma triggers a complex systemic response characterized by tissue injury, hemorrhage, hypoperfusion, and systemic inflammation.

TIC is now recognized as a complex and dynamic hemostatic disorder arising from the combined effects of severe tissue injury and hemorrhagic shock [3,4]. Rather than representing a purely hypocoagulable state, TIC involves the simultaneous activation and dysregulation of multiple coagulation and fibrinolytic pathways, with temporal evolution from an early hemorrhagic phenotype toward a potentially prothrombotic state during later phases of resuscitation and recovery. The pathophysiological alterations associated with TIC develop rapidly after injury, often preceding therapeutic interventions. In the early phase, extensive tissue injury triggers systemic thrombin generation through activation of the tissue factor pathway, leading to widespread coagulation activation that may resemble disseminated intravascular coagulation (DIC)-like processes [3,4,5]. This early procoagulant response occurs concurrently with consumption of coagulation factors, fibrinogen, and platelets, ultimately contributing to impaired clot formation and uncontrolled bleeding. Hypoperfusion and shock play a central role in the initiation of early TIC by activating the protein C pathway, resulting in proteolytic degradation of critical coagulation cofactors, particularly factors Va and VIIIa [3,4,5]. At the same time, depletion of endogenous anticoagulant proteins, including antithrombin, further contributes to dysregulated thrombin generation and loss of physiologic hemostatic balance. Shock-induced endothelial injury [14] also contributes substantially to TIC pathophysiology through shedding of the endothelial glycocalyx, a key regulator of vascular permeability, inflammation, and anticoagulant activity. Glycocalyx degradation promotes capillary leak, amplifies endothelial dysfunction, and exacerbates both hemorrhage and systemic coagulation activation. Hyperfibrinolysis represents another hallmark feature of early TIC [15]. Reduced inhibition of fibrinolysis, largely mediated by suppression of plasminogen activator inhibitor-1 (PAI-1), results in excessive plasmin generation and accelerated fibrin clot degradation. Clinically, hyperfibrinolysis is associated with severe hemodynamic instability and is a strong predictor of early mortality from hemorrhage [15]. However, fibrinolytic activity in trauma is highly dynamic. While early hyperfibrinolysis contributes to uncontrolled bleeding, many patients subsequently transition to a state of fibrinolytic shutdown characterized by impaired fibrin breakdown and increased thrombotic risk. This later phenotype has been associated with venous thromboembolism, organ dysfunction, and adverse outcomes, highlighting the evolving nature of TIC over time. Platelet dysfunction further contributes to impaired hemostasis in trauma patients. Although platelet counts may initially remain within normal ranges, qualitative defects in platelet activation, adhesion, and aggregation are frequently present [16]. This functional impairment correlates with injury severity and shock burden and contributes significantly to defective clot stability and persistent bleeding. As resuscitation progresses, additional iatrogenic and environmental factors, including excessive crystalloid administration, hypothermia, metabolic acidosis, and dilutional coagulopathy, contribute to the development of late TIC. These factors impair enzymatic reactions within the coagulation cascade, worsen platelet dysfunction, and further reduce circulating coagulation factor concentrations. The interaction between early endogenous coagulation disturbances and later iatrogenic derangements results in a rapidly evolving and highly heterogeneous coagulopathic state. Importantly, while hemorrhage predominates during the initial phase after trauma, many patients subsequently develop a prothrombotic phenotype driven by persistent thrombin generation, fibrinolytic shutdown, endothelial dysfunction, and inflammation. Recognition of TIC as a dynamic process involving both bleeding and thrombosis has important implications for monitoring strategies and targeted hemostatic interventions throughout trauma resuscitation (Figure 1).

## 4. Clinical Impact and Epidemiology

The presence of TIC on hospital admission is a powerful and independent predictor of adverse outcomes in severely injured patients. Individuals presenting with TIC have substantially higher transfusion requirements, are more likely to require massive transfusion protocols and experience increased rates of multi-organ dysfunction and failure [17]. Reported mortality rates among patients with TIC range from approximately 40% to as high as 80% in the most severely injured populations, underscoring the profound clinical impact of early coagulopathy [17]. TIC is more frequently observed in patients with severe physiological derangement, including profound hypotension, marked metabolic acidosis reflected by an elevated base deficit, severe traumatic brain injury, penetrating mechanisms of injury, and extensive soft tissue damage [18]. The strong association between TIC and both injury severity and shock highlight its role as an early marker of systemic insult rather than a late complication of resuscitation. Importantly, early recognition of TIC and prompt initiation of targeted, hemostatic therapy have been associated with improved clinical outcomes. These findings emphasize the need for systematic and early assessment of coagulation status during the initial phases of trauma care, enabling timely, goal-directed interventions aimed at mitigating ongoing hemorrhage and preventing downstream complications.

## 5. Diagnostic Modalities in TIC

**Figure 2:**
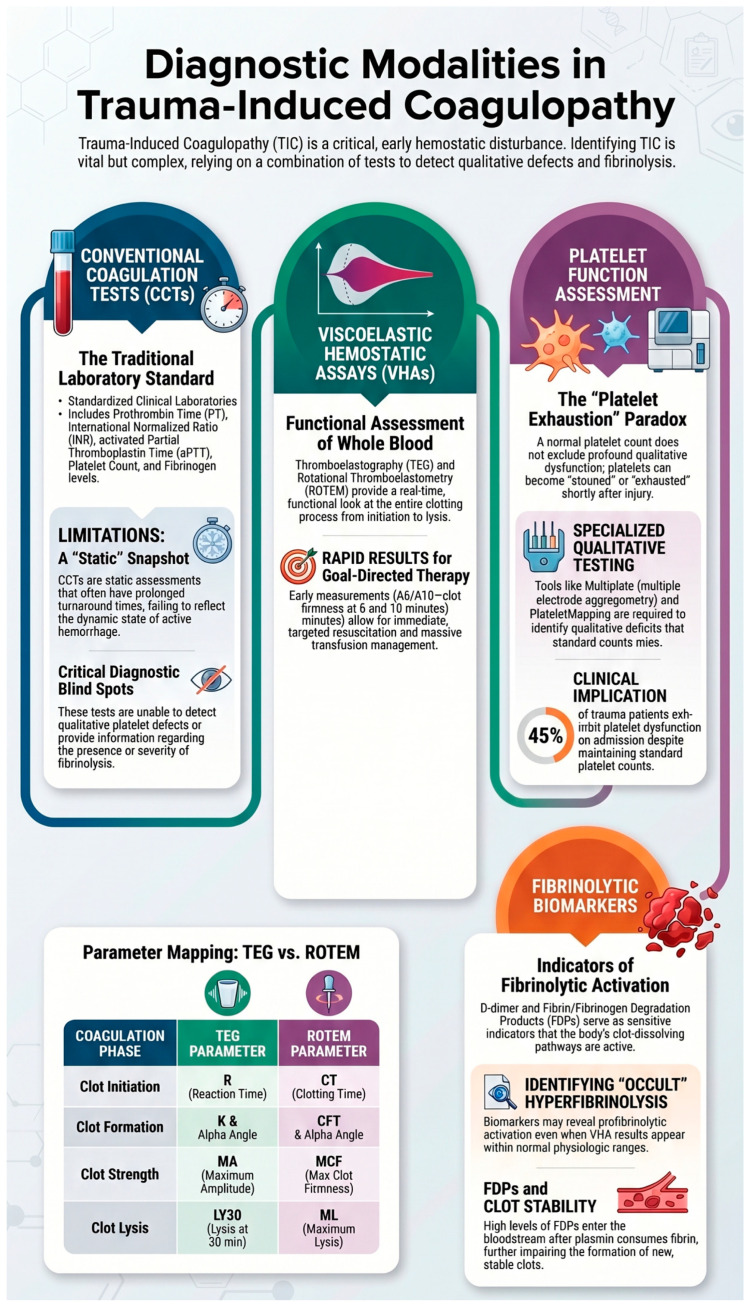
Diagnostic approach to TIC. The diagnostic workup of trauma patients integrates clinical assessment, conventional laboratory testing, and viscoelastic hemostatic assays. Standard laboratory parameters (including hemoglobin, platelet count, prothrombin time, activated partial thromboplastin time, fibrinogen levels, and base deficit) provide indirect assessment of coagulation status and tissue hypoperfusion. Viscoelastic tests such as thromboelastography (TEG) and rotational thromboelastometry (ROTEM) allow real-time, whole-blood evaluation of clot initiation, propagation, strength, and fibrinolysis, enabling rapid identification of specific coagulopathic phenotypes and guiding targeted transfusion strategies.

Conventional laboratory tests, including prothrombin time (PT), activated partial thromboplastin time (aPTT), fibrinogen concentration and platelet count, have long been used to assess coagulation status in trauma patients [5]. However, these assays have important limitations in the acute trauma setting. Turnaround times are often prolonged, and the results reflect isolated components of the coagulation cascade rather than the integrated, functional performance of whole blood. Moreover, conventional tests are insensitive to dynamic abnormalities such as impaired clot strength, platelet dysfunction, and hyperfibrinolysis, which are key features of trauma-induced coagulopathy [5]. Viscoelastic hemostatic testing has emerged as a valuable adjunct in the evaluation and management of TIC. Techniques such as rotational thromboelastometry (ROTEM) and thromboelastography (TEG) provide rapid, real-time, functional assessment of clot initiation, propagation, strength, and breakdown [19]. Unlike conventional coagulation assays, viscoelastic tests can rapidly identify major hemostatic abnormalities, including hypofibrinogenemia, impaired clot strength, and pathologic fibrinolysis, often within minutes of sample acquisition. These characteristics make viscoelastic testing particularly well suited for the acute management of bleeding trauma patients, enabling individualized, goal-directed resuscitation [19]. Several observational studies and growing clinical experience suggest that viscoelastic-guided resuscitation improves the precision of transfusion strategies, reduces unnecessary exposure to blood products, and facilitates earlier achievement of hemostasis [20]. Although high-quality randomized data remain limited, the integration of viscoelastic testing into trauma resuscitation algorithms has been increasingly endorsed by international guidelines to optimize hemostatic therapy in patients with severe hemorrhage (Figure 2).

However, although standard viscoelastic assays provide indirect information on platelet contribution to clot formation, they are limited in their ability to accurately assess qualitative platelet dysfunction. In trauma patients, platelet counts may remain within normal ranges despite substantial impairment in platelet activation, adhesion, and aggregation. Consequently, abnormalities in platelet function may not be fully captured by conventional viscoelastic assays alone. More advanced platelet-focused platforms, including TEG PlateletMapping and ROTEM platelet assays, may offer additional information; however, their clinical role in routine trauma resuscitation remains incompletely defined and they are not universally available. This limitation is clinically relevant, as trauma-associated platelet dysfunction has emerged as an important contributor to persistent bleeding and adverse outcomes despite apparently adequate clot strength on standard viscoelastic testing.

Therefore, viscoelastic assays should be interpreted within the broader clinical context and, when appropriate, integrated with conventional laboratory parameters and dedicated platelet function testing.

## 6. Principles of Hemostatic Resuscitation

The concept of hemostatic resuscitation reflects a shift toward early, targeted and coordinated management of hemorrhagic shock [21]. It is based on the understanding that minimizing hemodilution, correcting coagulopathy and restoring effective circulation must occur simultaneously [22]. Permissive hypotension seeks to maintain a level of blood pressure sufficient to preserve perfusion to vital organs while avoiding disruption of forming clots [23]. This approach is particularly relevant in penetrating trauma but is contraindicated in traumatic brain injury, where adequate cerebral perfusion must be maintained. Limiting crystalloid administration is a cornerstone of modern trauma care. Excessive infusion of crystalloids contributes to dilutional coagulopathy, exacerbates edema, induces hypothermia and worsens acidosis, collectively feeding into the lethal triad that historically contributed to poor outcomes. Contemporary guidelines [8] recommend minimizing crystalloid use in favor of early use of blood products. Damage control surgery complements hemostatic resuscitation by prioritizing rapid control of bleeding and contamination, followed by temporary stabilization rather than definitive repairs. This approach aims to interrupt the cycle of worsening shock, coagulopathy and hypothermia that often leads to a downward spiral if prolonged surgery is attempted in unstable patients [24].

## 7. Transfusion Strategies: Early Balanced Transfusion as the Cornerstone of Modern Trauma Resuscitation

Transfusion strategies in trauma have undergone a profound evolution over the past two decades, shifting from a sequential approach, traditionally based on the initial administration of red blood cells followed by delayed plasma and platelet supplementation, toward early, balanced resuscitation paradigms. This transition reflects an improved understanding of trauma-induced coagulopathy as an early and dynamic process requiring prompt correction. The PROPPR trial [11] provided high-level evidence supporting this paradigm shift. In this study, patients were randomized to receive transfusion ratios of either 1:1:1 or 1:1:2, where these ratios refer to the relative proportions of plasma, platelets, and red blood cells administered (i.e., one unit of each component in the 1:1:1 group versus a relative predominance of red blood cells in the 1:1:2 group). Although no significant difference in overall mortality was observed between the two strategies, patients receiving plasma, platelets, and red blood cells in a 1:1:1 ratio experienced fewer early deaths due to exsanguination and achieved hemostasis more frequently. These findings reinforce the concept that early, balanced administration of blood components mitigates the progression of coagulopathy, enhances hemostatic control, and improves early clinical outcomes. Collectively, this evidence has established balanced transfusion as the cornerstone of modern damage control resuscitation and provides the physiological and clinical foundation for the growing interest in whole blood-based resuscitation strategies.

Nevertheless, the use of fixed-ratio transfusion strategies has important limitations that should be acknowledged. Empirical balanced transfusion protocols may not adequately address the marked heterogeneity of coagulation abnormalities observed in trauma patients and may expose some patients to unnecessary plasma or platelet administration. In particular, fixed-ratio approaches may fail to sufficiently correct severe hypofibrinogenemia, which is one of the earliest and most clinically relevant coagulation abnormalities in major trauma. Because fresh frozen plasma contains relatively low concentrations of fibrinogen, large plasma volumes may be required to achieve meaningful fibrinogen replacement, potentially contributing to dilutional effects, volume overload, and delayed correction of critical fibrinogen deficiency. Accordingly, contemporary trauma guidelines increasingly recognize that fresh frozen plasma alone is often insufficient for the treatment of severe fibrinogen depletion and recommend early targeted fibrinogen supplementation using fibrinogen concentrate or cryoprecipitate in selected patients. In addition, growing evidence supports individualized hemostatic resuscitation guided by viscoelastic testing, allowing therapy to be tailored according to specific coagulation deficits rather than relying exclusively on empiric fixed-ratio transfusion protocols. Therefore, while balanced transfusion remains a central component of modern damage control resuscitation, current practice is progressively evolving toward more personalized, goal-directed approaches that integrate balanced component therapy with early coagulation monitoring and targeted factor replacement. This evolving paradigm has also provided part of the physiological and clinical rationale underlying renewed interest in whole blood-based resuscitation strategies.

Within this evolving framework, renewed interest has emerged in whole blood-based resuscitation strategies, particularly the use of low-titer group O whole blood (LTOWB). Whole blood offers several theoretical and practical advantages compared with conventional component therapy, including delivery of red blood cells, plasma, platelets, and coagulation factors in physiologic proportions within a single product, reduced exposure to additives and anticoagulants, lower donor exposure, and simplified transfusion logistics. Military experience during recent conflicts has further contributed to the resurgence of whole blood transfusion, particularly in settings characterized by massive hemorrhage and limited logistical resources [25]. Several retrospective civilian and military studies have suggested that whole blood transfusion may improve early hemostatic resuscitation and reduce blood product utilization; however, the available evidence remains heterogeneous and largely observational [25,26]. Systematic reviews and meta-analyses comparing whole blood with balanced component therapy have not consistently demonstrated a clear mortality benefit, largely due to variability in study design, patient populations, transfusion protocols, and types of whole blood used [25,26]. Furthermore, important practical limitations remain, including restricted product availability, storage constraints, concerns regarding ABO compatibility and alloimmunization, and variability in platelet preservation during storage. Consequently, although whole blood represents a promising strategy within modern damage control resuscitation, current evidence remains insufficient to establish clear superiority over optimized component-based therapy. Ongoing research is expected to better define which trauma populations may derive the greatest benefit from whole blood transfusion and how it can be most effectively integrated with goal-directed hemostatic resuscitation strategies.

## 8. Fibrinogen Replacement

Fibrinogen plays a central role in clot formation and hypofibrinogenemia is a common and early manifestation of trauma-induced coagulopathy [27]. During ongoing hemorrhage, fibrinogen levels decline rapidly, often reaching critically low thresholds before significant depletion of other coagulation factors occurs. Standard plasma contains relatively low fibrinogen concentrations, approximately 2 g per liter, necessitating large volumes to achieve meaningful hemostatic correction [27,28]. This limitation underscores the need for concentrated fibrinogen replacement strategies in the management of bleeding trauma patients. Fibrinogen concentrate offers several practical advantages, including rapid availability, precise dosing, and predictable enhancement of clot firmness [27,28]. Evidence from observational studies indicates that timely fibrinogen replacement improves clot mechanical properties and reduces ongoing hemorrhage. Cryoprecipitate, while effective, requires thawing and may not be immediately accessible in all clinical settings, limiting its utility in time-critical situations. Viscoelastic hemostatic assays, such as the FIBTEM test in rotational thromboelastometry, provide a functional assessment of fibrinogen contribution to clot strength and can guide targeted replacement therapy, as low FIBTEM amplitudes strongly correlate with hypofibrinogenemia [28]. The optimal timing and modality of fibrinogen replacement in trauma remain active areas of investigation. Some trauma centers have incorporated early administration of fibrinogen concentrate into first-line hemostatic resuscitation protocols, whereas others use a viscoelastic-guided approach to determine the need for replacement [29,30,31]. Although definitive randomized controlled trials are limited, the biological rationale and accumulating observational evidence support early correction of fibrinogen deficits as a key component of modern trauma resuscitation.

## 9. Coagulation Factor Therapy

Prothrombin complex concentrates (PCC) have gained increasing attention in trauma settings [32]. PCC provides rapid replacement of vitamin K-dependent coagulation factors and is well established for the urgent reversal of warfarin-associated coagulopathy. Compared with fresh frozen plasma, PCC allows faster correction of coagulation factor deficiencies while avoiding the delays associated with plasma thawing and administration of large transfusion volumes.

However, the role of PCC in trauma patients without preexisting anticoagulant exposure remains less clearly defined. Although prolonged prothrombin time (PT) or elevated international normalized ratio (INR) may reflect trauma-induced coagulopathy, these conventional laboratory abnormalities alone are not considered sufficient surrogate markers for PCC administration in bleeding trauma patients. TIC is a complex and heterogeneous disorder involving variable degrees of thrombin generation, fibrinogen depletion, platelet dysfunction, fibrinolytic abnormalities, and factor consumption, which may not be adequately characterized by PT/INR measurements alone. Consequently, indiscriminate PCC administration based solely on PT prolongation may expose patients to unnecessary thrombotic risk without effectively addressing the predominant hemostatic deficit.

Current evidence therefore supports a more individualized approach to PCC use, ideally guided by viscoelastic testing, evidence of delayed thrombin generation, persistent coagulopathy despite appropriate plasma and fibrinogen replacement, or specific clinical scenarios such as anticoagulant reversal [33,34,35,36]. In addition, concerns regarding thromboembolic complications persist, particularly in trauma patients who may subsequently transition toward a hypercoagulable phenotype during later phases of resuscitation.

Recombinant activated factor VII (rFVIIa) has also been explored as a hemostatic adjunct in trauma [37]. Although rFVIIa may enhance thrombin generation and improve clot formation, routine use is not recommended because randomized studies have failed to demonstrate consistent survival benefit, while concerns regarding arterial and venous thromboembolic complications remain significant. Current guidelines therefore reserve rFVIIa as a rescue therapy for selected patients with life-threatening hemorrhage that persists despite definitive surgical control, correction of hypothermia and acidosis, and optimization of other coagulation abnormalities [38,39].

## 10. Antifibrinolytic Therapy

The role of tranexamic acid in trauma was firmly established by the CRASH-2 trial [7], which demonstrated that early administration, within three hours of injury, reduces death from bleeding. The benefit is greatest when administered as soon as possible after injury, while administration after three hours may be harmful. Tranexamic acid acts by inhibiting plasminogen activation and slowing the breakdown of fibrin clots, thereby counteracting hyperfibrinolysis, one of the central mechanisms of TIC. In traumatic brain injury, the CRASH-3 trial [10] provided evidence that early administration of TXA reduces head injury-related mortality in patients with mild to moderate TBI. These findings underscore the importance of early recognition and prompt treatment of fibrinolytic abnormalities in trauma.

However, a major unresolved limitation in the clinical use of antifibrinolytic therapy is the inability to reliably identify which patients truly exhibit clinically relevant hyperfibrinolysis at the time of presentation. Conventional viscoelastic assays such as TEG and ROTEM have limited sensitivity for detecting fibrinolytic activation, as hyperfibrinolysis is often transient, spatially heterogeneous, and may occur below the detection threshold of standard assays. As a result, clinically significant fibrinolytic activity may go unrecognized, while TXA continues to be administered in a largely empiric and non-individualized manner. In this context, emerging diagnostic strategies such as tissue plasminogen activator (tPA)-augmented viscoelastic assays represent a promising development toward functional, patient-specific assessment of fibrinolytic potential. By exogenously challenging the coagulation system with tPA, these modified assays unmask a latent susceptibility to fibrinolysis that may not be apparent under baseline conditions, thereby providing a more sensitive and dynamic evaluation of fibrinolytic reserve. This approach may help to better stratify patients at risk of dysregulated fibrinolysis and ultimately refine indications, timing, and dosing of tranexamic acid. Recent evidence supporting this concept and its potential clinical implications is discussed in detail by Moore et al. [40], who highlight how tPA-modified assays could bridge the gap between empiric antifibrinolytic therapy and precision-guided haemostatic resuscitation, although clinical validation and integration into trauma algorithms remain ongoing challenges.

## 11. Damage Control Surgery and Endovascular Approaches

Damage control surgery (DCS) consists of abbreviated operative procedures aimed at rapid haemostasis and contamination control in physiologically unstable patients, thereby limiting operative time and preventing progression of coagulopathy, hypothermia, and acidosis. This staged approach, including techniques such as temporary abdominal closure, packing, and rapid control of major injuries, improves survival in exsanguinating hemorrhage, particularly when integrated with early hemostatic resuscitation [21,22]. Endovascular techniques provide complementary options for selected sources of non-compressible hemorrhage. Resuscitative endovascular balloon occlusion of the aorta (REBOA) may be used as a temporary adjunct to achieve proximal hemorrhage control and maintain perfusion to the heart and brain; however, its use requires strict patient selection, significant operator expertise, and careful consideration of occlusion time due to the risk of distal ischemia and reperfusion injury. Importantly, REBOA should be regarded as a bridge to definitive surgical or endovascular hemorrhage control rather than a definitive therapy. Targeted angioembolization represents an established minimally invasive option for definitive bleeding control in pelvic, solid organ, and selected vascular injuries, contingent on rapid access to interventional radiology and multidisciplinary coordination [41,42,43,44].

Overall, modern hemorrhage control strategies should integrate DCS and endovascular techniques within a physiology-driven framework, where early procedural intervention is combined with hemostatic resuscitation and viscoelastic-guided transfusion to optimize survival while minimizing progression of trauma-induced coagulopathy.

## 12. Integration of Viscoelastic Diagnostics into Hemostatic Resuscitation

Viscoelastic testing (VET) enables an individualized approach to trauma resuscitation [20,45]. Rather than applying uniform transfusion ratios, clinicians can tailor therapy to correct specific deficits. When low fibrinogen levels are detected, early administration of fibrinogen concentrate can restore clot firmness. If clot amplitude is reduced despite adequate fibrinogen, platelet dysfunction may require platelet transfusion. Prolonged clotting times may signal factor deficiencies that can be treated with plasma or PCC. Excessive clot breakdown requires antifibrinolytic therapy. Several studies have shown that VET-guided therapy can reduce the number of blood products administered, support faster correction of coagulopathy, and improve outcomes. Although implementation varies internationally, viscoelastic diagnostics represent a cornerstone of contemporary hemostatic resuscitation where available [46,47,48,49,50].

However, the interpretation of VET parameters is not always directly translatable into effective correction of in vivo haemostasis, particularly in the case of PCC-guided strategies. Recent evidence has highlighted that VET-derived clotting time parameters only partially reflect thrombin generation capacity, and that PCC administration guided exclusively by these thresholds may result in variable and sometimes insufficient restoration of haemostatic potential, raising concerns about potential overtreatment without consistent physiological benefit [49]. These limitations underscore a fundamental disconnect between simplified viscoelastic surrogates and the complexity of enzymatic coagulation.

To address this gap, recently proposed algorithm-based approaches derived from large real-life trauma cohorts have moved beyond isolated parameter correction toward integrated, stepwise haemostatic protocols. These contemporary algorithms typically combine sequential decision nodes based on VET variables (e.g., fibrin-based clot strength, clotting time, and fibrinolysis indices) with predefined thresholds that trigger specific interventions in a structured order—most commonly fibrinogen replacement first, followed by platelet therapy if clot amplitude remains reduced, and selective use of plasma or PCC only in cases of persistent clotting time prolongation after correction of fibrinogen and platelet deficits. Antifibrinolytic therapy is incorporated early when fibrinolytic activation is detected or strongly suspected. In addition, these protocols emphasize reassessment after each intervention, reflecting the dynamic nature of trauma-induced coagulopathy rather than a single-point decision model. Such structured, algorithm-driven VET-guided strategies have been shown in recent cohort-based studies to improve standardization of haemostatic resuscitation and reduce variability in transfusion practice, while maintaining individualized treatment principles in real-world trauma settings [50]. Importantly, these approaches also implicitly acknowledge the limitations of PCC responsiveness within VET frameworks, restricting its use to more refractory or persistent coagulation factor deficits rather than early or isolated clotting time abnormalities.

Overall, VET remains a cornerstone of contemporary haemostatic resuscitation; however, its optimal use increasingly depends on structured, algorithm-based interpretation rather than isolated parameter-driven decision-making, particularly with respect to the cautious and context-dependent use of PCC.

## 13. Clinical Guidelines and Consensus Recommendations

Guidelines from major international trauma organizations consistently define the core principles of modern hemostatic resuscitation, providing formal recommendations for the early management of trauma-induced coagulopathy (TIC). Across these guidelines, there is strong agreement that initial management should include rapid identification of patients at risk of TIC, immediate administration of tranexamic acid in eligible patients within the recommended time window, and early initiation of balanced transfusion strategies using plasma, platelets, and red blood cells in appropriate ratios. In addition, both the European and international guideline frameworks formally recommend early correction of hypofibrinogenemia, either with fibrinogen concentrate or cryoprecipitate, when laboratory or viscoelastic evidence of reduced clot strength is present. The use of viscoelastic testing (TEG or ROTEM) is explicitly endorsed in several guidelines as a preferred tool to guide goal-directed transfusion and to individualize haemostatic therapy in bleeding trauma patients. Formal recommendations also consistently address adjunctive principles of damage control resuscitation. These include minimization of crystalloid administration, prevention of hypothermia and acidosis, and early implementation of damage control surgery as part of a coordinated strategy to prevent progression of coagulopathy and physiological collapse. Regarding procedural haemorrhage control, guidelines recommend consideration of resuscitative endovascular balloon occlusion of the aorta (REBOA) and angioembolization only in selected patients with non-compressible haemorrhage, emphasizing that these techniques should be integrated within a multidisciplinary pathway and used as adjuncts rather than standalone interventions.

Several major guidelines provide detailed frameworks:

• European guideline (6th edition): Offers comprehensive recommendations on diagnostics, transfusion ratios, coagulation factor replacement, and integration of procedural interventions, emphasizing proactive management of coagulopathy [8].
• American College of Surgeons—Committee on Trauma (ACS-COT): Recommends early balanced transfusion, massive transfusion protocols, antifibrinolytic therapy, and the incorporation of damage control surgery and hemostatic resuscitation principles into ATLS algorithms [51].
• Eastern Association for the Surgery of Trauma (EAST): Provides evidence-based guidance for transfusion strategies, factor replacement (fibrinogen, PCC), use of rFVIIa as rescue therapy, and VET-guided individualized management [52].
• World Society of Emergency Surgery (WSES): Advocates a global, physiology-driven approach integrating systemic hemostatic resuscitation, procedural hemorrhage control, viscoelastic diagnostics, and stepwise factor replacement [9].

## 14. Special Considerations in Subpopulations

The principles of hemostatic resuscitation apply broadly across trauma populations, but certain subgroups, such as patients with traumatic brain injury (TBI), geriatric patients and pediatric patients, require tailored approaches to optimize outcomes. In patients with TBI, permissive hypotension is generally contraindicated, as maintaining adequate cerebral perfusion pressure is essential to prevent secondary brain injury. Early administration of tranexamic acid (TXA) appears to confer benefit in patients with mild to moderate TBI, reducing hemorrhagic progression and mortality, whereas its efficacy in severe TBI is less clear, and timing of administration is critical, ideally within the first 3 h of injury [53,54,55]. Older adults often present with comorbidities and may be receiving anticoagulant or antiplatelet therapy, which complicates hemostasis and increases the risk of hemorrhage. In these patients, rapid reversal protocols are paramount. Prothrombin complex concentrates (PCC) are recommended for vitamin K antagonists, idarucizumab for dabigatran, and andexanet alfa for factor Xa inhibitors. Timely reversal, in conjunction with targeted hemostatic resuscitation, can mitigate bleeding complications and improve outcomes [56,57]. Children exhibit distinct physiological responses to hemorrhage, including a more rapid decline in fibrinogen levels and increased sensitivity to hypovolemia. Additionally, evidence guiding transfusion ratios, factor replacement, and viscoelastic monitoring in children is more limited, necessitating careful extrapolation from adult data and individualized clinical decision-making [58,59,60,61,62,63]. Tailoring hemostatic resuscitation strategies to these subgroups, considering age, preexisting comorbidities, anticoagulation status, and type of injury, enhances the effectiveness of early interventions and supports improved survival across diverse trauma populations.

## 15. Discussion

Despite major advances in the understanding and management of trauma-induced coagulopathy (TIC), its clinical translation remains constrained by the difficulty of capturing a rapidly evolving and highly heterogeneous haemostatic state with currently available diagnostic tools. In practice, most therapeutic strategies still rely on surrogate markers that only partially reflect the underlying biology, which can lead to a degree of empiricism even within otherwise structured resuscitation pathways. This is particularly relevant in the earliest phases of trauma care, where time-critical decisions must often be made before a stable and complete haemostatic profile can be established.

Although viscoelastic testing has clearly improved bedside assessment of coagulation, important limitations persist in its ability to resolve the full complexity of trauma-related haemostatic derangements. Certain clinically relevant phenotypes, particularly those involving subtle fibrinolytic activation or qualitative platelet dysfunction, may remain undetected or underestimated. This diagnostic uncertainty inevitably influences treatment decisions and contributes to variability in clinical practice, despite the apparent standardization introduced by point-of-care testing.

A similar issue emerges when considering factor-based therapies, especially prothrombin complex concentrates. While viscoelastic parameters provide a practical framework for guiding therapy, they do not always correspond to actual in vivo thrombin generation, which raises concerns about the biological adequacy of correction in some patients. This gap between laboratory improvement and true physiological restoration remains one of the central unresolved issues in goal-directed haemostatic resuscitation, and it complicates the definition of optimal thresholds for intervention.

Antifibrinolytic therapy is affected by a related problem: the inability to reliably and consistently identify patients with clinically significant fibrinolysis at presentation. Standard viscoelastic assays are often insensitive to low-grade or transient fibrinolytic activity, which may still be clinically relevant. As a result, treatment decisions with tranexamic acid continue to rely largely on early empirical administration rather than precise phenotypic stratification, despite increasing recognition that fibrinolysis is not a uniform or binary phenomenon.

More recently, algorithm-based approaches derived from real-world trauma cohorts have attempted to reduce variability by structuring viscoelastic-guided therapy into reproducible decision pathways. These represent an important step toward standardization, but they still largely depend on fixed thresholds and sequential logic that may not fully reflect the continuous and adaptive nature of coagulation dynamics in vivo. Their external validity also remains limited by heterogeneity in patient populations, institutional protocols, and resource availability.

Against this background, future progress is likely to depend on moving beyond single-modality assessment toward more integrated and dynamic models of haemostasis. This includes the development of more sensitive functional assays capable of probing fibrinolytic reserve and thrombin generation under physiological challenge, as well as the incorporation of multimodal data streams that extend beyond clot-based metrics alone. In parallel, data-driven approaches such as machine learning may help to better integrate complex physiological signals and support earlier identification of haemostatic phenotypes that are not readily apparent with current tools.

**Figure 3:**
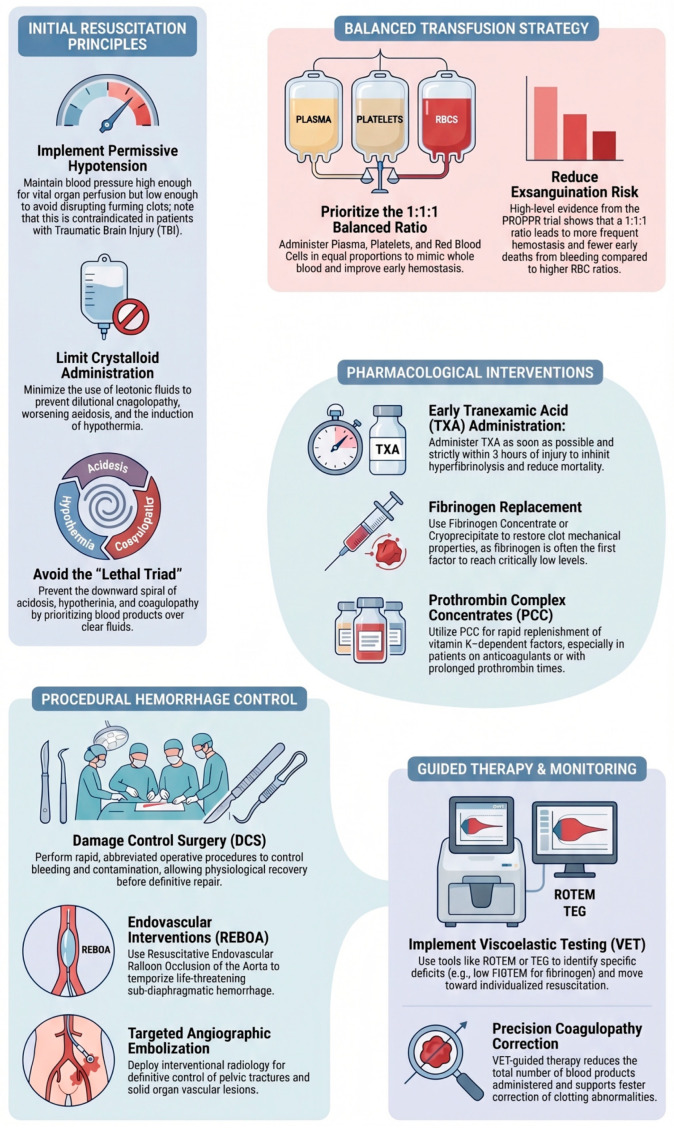
Therapeutic strategies for trauma-induced coagulopathy (TIC). Management of trauma-induced coagulopathy is based on early, goal-directed resuscitation aimed at controlling hemorrhage and restoring hemostatic competence. Initial treatment includes rapid hemorrhage control and implementation of damage control resuscitation principles, with early administration of blood products using either balanced component therapy (e.g., 1:1:1 ratio of plasma, platelets, and red blood cells) or whole blood when available. Adjunctive therapies include early administration of antifibrinolytic agents such as tranexamic acid, fibrinogen supplementation (via cryoprecipitate or fibrinogen concentrate), and targeted replacement of coagulation factors guided by viscoelastic testing (e.g., TEG or ROTEM). Correction of contributing factors such as hypothermia, acidosis, and hypocalcemia is essential to optimize coagulation function. Advanced strategies may include the use of prothrombin complex concentrates in selected cases. A multimodal, individualized approach is required to rapidly reverse coagulopathy, limit ongoing bleeding, and improve clinical outcomes.

Ultimately, the major unmet need remains the transition from protocol-driven to truly adaptive and patient-specific resuscitation strategies. Achieving this will require not only improved diagnostics, but also prospective validation of individualized treatment algorithms with outcomes that extend beyond transfusion efficiency to include organ dysfunction, thrombotic risk, and long-term recovery. Figure 3 is a summary of therapeutic approaches for hemostatic resuscitation in TIC.

## 16. Conclusions

Trauma-induced coagulopathy represents a major driver of morbidity and mortality in severely injured patients, and its early recognition and correction are essential to improving outcomes. Hemostatic resuscitation combines a series of coordinated interventions aimed at restoring effective hemostasis, including balanced transfusion, early fibrinogen replacement, targeted factor therapy, antifibrinolytic treatment, and minimal crystalloid administration. Complemented by permissive hypotension, damage control surgery, and viscoelastic-guided therapy, this approach has transformed trauma care and reduced preventable deaths. Continued refinement and broader implementation of these strategies, guided by emerging evidence and technological advances, will be essential for further improving survival after severe trauma.

## Data Availability

The data used to support the findings of this study are included within the article. The data presented in this study are available on request.

## References

[1] Kauvar D.S., Lefering R., Wade C.E. (2006). Impact of hemorrhage on trauma outcome: An overview of epidemiology, clinical presentations, and therapeutic considerations. J. Trauma Inj. Infect. Crit. Care.

[2] Brohi K., Singh J., Heron M., Coats T. (2003). Acute traumatic coagulopathy. J. Trauma Inj. Infect. Crit. Care.

[3] Brohi K., Cohen M.J., Davenport R.A. (2007). Acute coagulopathy of trauma: Mechanism, identification and effect. Curr. Opin. Crit. Care.

[4] Hess J.R., Brohi K., Dutton R.P., Hauser C.J., Holcomb J.B., Kluger Y., Mackway-Jones K., Parr M.J., Rizoli S.B., Yukioka T. (2008). The coagulopathy of trauma: A review of mechanisms. J. Trauma Inj. Infect. Crit. Care.

[5] Davenport R., Manson J., De’Ath H., Platton S., Coates A., Allard S., Hart D., Pearse R., Pasi K.J., MacCallum P. (2011). Functional definition and characterization of acute traumatic coagulopathy. Crit. Care Med..

[6] Nguyen M., Pirracchio R., Kornblith L.Z., Callcut R., Fox E.E., Wade C.E., Schreiber M., Holcomb J.B., Coyle J., Cohen M. (2020). Dynamic impact of transfusion ratios on outcomes in severely injured patients: Targeted machine learning analysis of the Pragmatic, Randomized Optimal Platelet and Plasma Ratios randomized clinical trial. J. Trauma Acute Care Surg..

[7] Shakur H., Roberts I., Bautista R., Caballero J., Coats T., Dewan Y., El-Sayed H., Gogichaishvili T., Gupta S., CRASH-2 Trial Collaborators (2010). Effects of tranexamic acid on death, vascular occlusive events, and blood transfusion in trauma patients with significant haemorrhage (CRASH-2): A randomised, placebo-controlled trial. Lancet.

[8] Rossaint R., Afshari A., Bouillon B., Cerny V., Cimpoesu D., Curry N., Duranteau J., Filipescu D., Grottke O., Grønlykke L. (2023). The European guideline on management of major bleeding and coagulopathy following trauma: Sixth edition. Crit. Care.

[9] König C., Adam G., Well L. (2025). Management von Organblutungen: Leitlinien der American Association for the Surgery of Trauma (AAST) und World Society of Emergency Surgery (WSES) [Management of internal bleeding: Guidelines of the American Association for the Surgery of Trauma (AAST) and World Society of Emergency Surgery (WSES)]. Radiologie.

[10] CRASH-3 Trial Collaborators (2019). Effects of tranexamic acid on death, disability, vascular occlusive events and other morbidities in patients with acute traumatic brain injury (CRASH-3): A randomised, placebo-controlled trial. Lancet.

[11] Holcomb J.B., Tilley B.C., Baraniuk S., Fox E.E., Wade C.E., Podbielski J.M., del Junco D.J., Brasel K.J., Bulger E.M., Callcut R.A. (2015). Transfusion of plasma, platelets, and red blood cells in a 1:1:1 vs a 1:1:2 ratio and mortality in patients with severe trauma: The PROPPR randomized clinical trial. JAMA.

[12] Sperry J.L., Guyette F.X., Brown J.B., Yazer M.H., Triulzi D.J., Early-Young B.J., Adams P.W., Daley B.J., Miller R.S., Harbrecht B.G. (2018). Prehospital Plasma during Air Medical Transport in Trauma Patients at Risk for Hemorrhagic Shock. N. Engl. J. Med..

[13] Moore H.B., Moore E.E., Chapman M.P., McVaney K., Bryskiewicz G., Blechar R., Chin T., Burlew C.C., Pieracci F., West F.B. (2018). Plasma-first resuscitation to treat haemorrhagic shock during emergency ground transportation in an urban area: A randomised trial. Lancet.

[14] Johansson P.I., Stensballe J., Ostrowski S.R. (2017). Shock induced endotheliopathy (SHINE) in acute critical illness—A unifying pathophysiologic mechanism. Crit. Care.

[15] Moore H.B., Moore E.E., Gonzalez E., Chapman M.P., Chin T.L., Silliman C.C., Banerjee A., Sauaia A. (2014). Hyperfibrinolysis, physiologic fibrinolysis, and fibrinolysis shutdown: The spectrum of postinjury fibrinolysis and relevance to antifibrinolytic therapy. J. Trauma Acute Care Surg..

[16] Kutcher M.E., Redick B.J., McCreery R.C., Crane I.M., Greenberg M.D., Cachola L.M., Nelson M.F., Cohen M.J. (2012). Characterization of platelet dysfunction after trauma. J. Trauma Acute Care Surg..

[17] MacLeod J.B., Lynn M., McKenney M.G., Cohn S.M., Murtha M. (2003). Early coagulopathy predicts mortality in trauma. J. Trauma Acute Care Surg..

[18] Brohi K., Cohen M.J., Ganter M.T., Schultz M.J., Levi M., Mackersie R.C., Pittet J.F. (2008). Acute coagulopathy of trauma: Hypoperfusion induces systemic anticoagulation and hyperfibrinolysis. J. Trauma Inj. Infect. Crit. Care.

[19] Whiting D., DiNardo J.A. (2014). TEG and ROTEM: Technology and clinical applications. Am. J. Hematol..

[20] Moore E.E., Moore H.B., Chapman M.P., Gonzalez E., Sauaia A. (2018). Goal-directed hemostatic resuscitation for trauma induced coagulopathy: Maintaining homeostasis. J. Trauma Acute Care Surg..

[21] Rotondo M.F., Schwab C.W., McGonigal M.D., Phillips G.R., Fruchterman T.M., Kauder D.R., Latenser B.A., Angood P.A. (1993). ‘Damage control’: An approach for improved survival in exsanguinating penetrating abdominal injury. J. Trauma Acute Care Surg..

[22] Holcomb J.B., Jenkins D., Rhee P., Johannigman J., Mahoney P., Mehta S., Cox E.D., Gehrke M.J., Beilman G.J., Schreiber M. (2007). Damage control resuscitation: Directly addressing the early coagulopathy of trauma. J. Trauma Inj. Infect. Crit. Care.

[23] Bickell W.H., Wall M.J., Pepe P.E., Martin R.R., Ginger V.F., Allen M.K., Mattox K.L. (1994). Immediate versus delayed fluid resuscitation for hypotensive patients with penetrating torso injuries. N. Engl. J. Med..

[24] Gunter O.L., Au B.K., Isbell J.M., Mowery N.T., Young P.P., Cotton B.A. (2008). Optimizing outcomes in damage control resuscitation: Identifying blood product ratios associated with improved survival. J. Trauma Inj. Infect. Crit. Care.

[25] Ibrahim W., Meza Monge K., Menzel J., Morales Ojeda L., Dalton M., Fuenmayor Lozada D.A., Vidal-Gallardo A., Fonseca Niño A.M., Aragón Conrado L.E., Cripps M.W. (2026). Whole-Blood vs Component Therapy in Adult Trauma: An Updated Systematic Review and Meta-Analysis. JAMA Surg..

[26] Meizoso J.P., Cotton B.A., Lawless R.A., Kodadek L.M., Lynde J.M., Russell N., Gaspich J., Maung A., Anderson C., Reynolds J.M. (2024). Whole blood resuscitation for injured patients requiring transfusion: A systematic review, meta-analysis, and practice management guideline from the Eastern Association for the Surgery of Trauma. J. Trauma Acute Care Surg..

[27] Fries D., Martini W.Z. (2010). Role of fibrinogen in trauma-induced coagulopathy. Br. J. Anaesth..

[28] Davenport R., Curry N. (2024). Fibrinogen replacement in trauma haemorrhage: Essential but not empirical?. Emerg. Med. J..

[29] Burt T., Guilliam A., Cole E., Davenport R. (2025). Effect of early administration of fibrinogen replacement therapy in traumatic haemorrhage: A systematic review and meta-analysis of randomised controlled trials with narrative synthesis of observational studies. Crit. Care.

[30] Curry N., Foley C., Wong H., Mora A., Curnow E., Zarankaite A., Hodge R., Hopkins V., Deary A., Ray J. (2018). Early fibrinogen concentrate therapy for major haemorrhage in trauma (E-FIT 1): Results from a UK multi-centre, randomised, double blind, placebo-controlled pilot trial. Crit. Care.

[31] Steinmetz J., Sørensen A.M., Henriksen H.H., Lange T., Larsen C.F., Johansson P.I., Stensballe J. (2016). Pilot Randomized trial of Fibrinogen in Trauma Haemorrhage (PRooF-iTH): Study protocol for a randomized controlled trial. Trials.

[32] Baksaas-Aasen K., Gall L., Eaglestone S., Rourke C., Juffermans N.P., Goslings J.C., Naess P.A., van Dieren S., Ostrowski S.R., Stensballe J. (2017). iTACTIC—Implementing Treatment Algorithms for the Correction of Trauma-Induced Coagulopathy: Study protocol for a multicentre, randomised controlled trial. Trials.

[33] Franchini M., Lippi G. (2010). Prothrombin complex concentrates: An update. Blood Transfus..

[34] Grottke O., Heubner L. (2025). Restoring hemostasis with prothrombin complex concentrate: Benefits and risks in trauma-induced coagulopathy. Curr. Opin. Anaesthesiol..

[35] Matsushima K., Benjamin E., Demetriades D. (2015). Prothrombin complex concentrate in trauma patients. Am. J. Surg..

[36] Kao T.W., Lee Y.C., Chang H.T. (2021). Prothrombin Complex Concentrate for Trauma Induced Coagulopathy: A Systematic Review and Meta-Analysis. J. Acute Med..

[37] Hannadjas I., James A., Davenport R., Lindsay C., Brohi K., Cole E. (2023). Prothrombin complex concentrate (PCC) for treatment of trauma-induced coagulopathy: Systematic review and meta-analyses. Crit. Care.

[38] Dutton R.P., Conti B.M. (2009). The role of recombinant-activated factor VII in bleeding trauma patients. Curr. Opin. Anaesthesiol..

[39] Bartal C., Yitzhak A. (2009). The role of thromboelastometry and recombinant factor VIIa in trauma. Curr. Opin. Anaesthesiol..

[40] Duchesne J.C., Mathew K.A., Marr A.B., Pinsky M.R., Barbeau J.M., McSwain N.E. (2008). Current evidence based guidelines for factor VIIa use in trauma: The good, the bad, and the ugly. Am. Surg..

[41] Morrison J.J., Galgon R.E., Jansen J.O., Cannon J.W., Rasmussen T.E., Eliason J.L. (2016). A systematic review of the use of resuscitative endovascular balloon occlusion of the aorta in the management of hemorrhagic shock. J. Trauma Acute Care Surg..

[42] Borger van der Burg B.L.S., van Dongen T.T.C.F., Morrison J.J., Hedeman Joosten P.P.A., DuBose J.J., Hörer T.M., Hoencamp R. (2018). A systematic review and meta-analysis of the use of resuscitative endovascular balloon occlusion of the aorta in the management of major exsanguination. Eur. J. Trauma Emerg. Surg..

[43] Granieri S., Frassini S., Cimbanassi S., Bonomi A., Paleino S., Lomaglio L., Chierici A., Bruno F., Biondi R., Di Saverio S. (2022). Impact of resuscitative endovascular balloon occlusion of the aorta (REBOA) in traumatic abdominal and pelvic exsanguination: A systematic review and meta-analysis. Eur. J. Trauma Emerg. Surg..

[44] Brenner M., Teeter W., Hoehn M., Pasley J., Hu P., Yang S., Romagnoli A., Diaz J., Stein D., Scalea T. (2018). Use of Resuscitative Endovascular Balloon Occlusion of the Aorta for Proximal Aortic Control in Patients With Severe Hemorrhage and Arrest. JAMA Surg..

[45] Petrone P., Pérez-Jiménez A., Rodríguez-Perdomo M., Brathwaite C.E.M., Joseph D.K. (2019). Resuscitative Endovascular Balloon Occlusion of the Aorta (REBOA) in the Management of Trauma Patients: A Systematic Literature Review. Am. Surg..

[46] Stensballe J., Henriksen H.H., Johansson P.I. (2017). Early haemorrhage control and management of trauma-induced coagulopathy: The importance of goal-directed therapy. Curr. Opin. Crit. Care.

[47] Longacre M.M., Ibla J.C. (2025). TEG and ROTEM: Technology and Clinical Applications, 2026 Update. Am. J. Hematol..

[48] Whiting P., Al M., Westwood M., Ramos I.C., Ryder S., Armstrong N., Misso K., Ross J., Severens J., Kleijnen J. (2015). Viscoelastic point-of-care testing to assist with the diagnosis, management and monitoring of haemostasis: A systematic review and cost-effectiveness analysis. Health Technol. Assess..

[49] Brill J.B., Brenner M., Duchesne J., Roberts D., Ferrada P., Horer T., Kauvar D., Khan M., Kirkpatrick A., Ordonez C. (2021). The Role of TEG and ROTEM in Damage Control Resuscitation. Shock.

[50] Hofmann N., Schöchl H., Zipperle J., Gratz J., Schmitt F.C.F., Oberladstätter D. (2025). Altered thrombin generation with prothrombin complex concentrate is not detected by viscoelastic testing: An in vitro study. Br. J. Anaesth..

[51] Hofmann N., Schöchl H., Oberladstätter D., Zipperle J., Schmitt F., von der Forst M., Martin C., Gratz J. (2026). Viscoelastic coagulation testing in bleeding trauma patients: A retrospective analysis and development of a treatment algorithm. Br. J. Anaesth..

[52] American College of Surgeons Committee on Trauma (2018). Advanced Trauma Life Support (ATLS®) Student Course Manual.

[53] Cannon J.W., Khan M.A., Raja A.S., Cohen M.J., Como J.J., Cotton B.A., Dubose J.J., Fox E.E., Inaba K., Rodriguez C.J. (2017). Damage control resuscitation in patients with severe traumatic hemorrhage: A practice management guideline from the Eastern Association for the Surgery of Trauma. J. Trauma Acute Care Surg..

[54] Rowell S.E., Meier E.N., McKnight B., Kannas D., May S., Sheehan K., Bulger E.M., Idris A.H., Christenson J., Morrison L.J. (2020). Effect of Out-of-Hospital Tranexamic Acid vs Placebo on 6-Month Functional Neurologic Outcomes in Patients With Moderate or Severe Traumatic Brain Injury. JAMA.

[55] Sprigg N., Flaherty K., Appleton J.P., Al-Shahi Salman R., Bereczki D., Beridze M., Ciccone A., Collins R., Dineen R.A., Duley L. (2019). Tranexamic acid to improve functional status in adults with spontaneous intracerebral haemorrhage: The TICH-2 RCT. Health Technol. Assess..

[56] Karl V., Thorn S., Mathes T., Hess S., Maegele M. (2022). Association of Tranexamic Acid Administration With Mortality and Thromboembolic Events in Patients with Traumatic Injury: A Systematic Review and Meta-analysis. JAMA Netw. Open.

[57] Levi M. (2016). Management of bleeding in patients treated with direct oral anticoagulants. Crit. Care..

[58] Kaatz S., Mahan C.E., Nakhle A., Gunasekaran K., Ali M., Lavender R., Paje D.G. (2017). Management of Elective Surgery and Emergent Bleeding with Direct Oral Anticoagulants. Curr. Cardiol. Rep..

[59] Perea L.L., Moore K., Docherty C., Nguyen U., Seamon M.J., Byrne J.P., Jenkins D.H., Braverman M.A., Porter J.M., Armento I.G. (2023). Whole Blood Resuscitation is Safe in Pediatric Trauma Patients: A Multicenter Study. Am. Surg..

[60] Feeney E.V., Morgan K.M., Spinella P.C., Gaines B.A., Leeper C.M. (2024). Whole blood: Total blood product ratio impacts survival in injured children. J. Trauma Acute Care Surg..

[61] Anand T., Obaid O., Nelson A., Chehab M., Ditillo M., Hammad A., Douglas M., Bible L., Joseph B. (2021). Whole blood hemostatic resuscitation in pediatric trauma: A nationwide propensity-matched analysis. J. Trauma Acute Care Surg..

[62] Abou Khalil E., Morgan K.M., Gaines B.A., Spinella P.C., Leeper C.M. (2024). Use of whole blood in pediatric trauma: A narrative review. Trauma Surg. Acute Care Open.

[63] Kornelsen E., Kuppermann N., Nishijima D.K., Ren L.Y., Rumantir M., Gill P.J., Finkelstein Y. (2022). Effectiveness and safety of tranexamic acid in pediatric trauma: A systematic review and meta-analysis. Am. J. Emerg. Med..

